# Effect of stemming on text similarity for Arabic language at sentence level

**DOI:** 10.7717/peerj-cs.530

**Published:** 2021-05-14

**Authors:** Mohammad O. Alhawarat, Hikmat Abdeljaber, Anwer Hilal

**Affiliations:** 1 Department of Computer Science, College of Computer Engineering and Sciences, Prince Sattam Bin Abdulaziz University, Alkharj, Saudi Arabia; 2 General Department, College of Preparatory Year, Prince Sattam Bin Abdulaziz University, Alkharj, Saudi Arabia

**Keywords:** Semantic text similarity, Natural language processing, Stemming, Lemmatization, Machine learning, Word embedding, TF-IDF

## Abstract

Semantic Text Similarity (STS) has several and important applications in the field of Natural Language Processing (NLP). The Aim of this study is to investigate the effect of stemming on text similarity for Arabic language at sentence level. Several Arabic light and heavy stemmers as well as lemmatization algorithms are used in this study, with a total of 10 algorithms. Standard training and testing data sets are used from SemEval-2017 international workshop for Task 1, Track 1 Arabic (ar–ar). Different features are selected to study the effect of stemming on text similarity based on different similarity measures. Traditional machine learning algorithms are used such as Support Vector Machines (SVM), Stochastic Gradient Descent (SGD) and Naïve Bayesian (NB). Compared to the original text, using the stemmed and lemmatized documents in experiments achieve enhanced Pearson correlation results. The best results attained when using Arabic light Stemmer (ARLSTem) and Farasa light stemmers, Farasa and Qalsadi Lemmatizers and Tashaphyne heavy stemmer. The best enhancement was about 7.34% in Pearson correlation. In general, stemming considerably improves the performance of sentence text similarly for Arabic language. However, some stemmers make results worse than those for original text; they are Khoja heavy stemmer and AlKhalil light stemmer.

## Introduction

STS is usually used to measure the similarity of the meaning between two words, sentences, or documents. STS appears to be a challenging task, especially for short text. It has important and several applications in the field of NLP and computational linguistics. Examples of applications are documents classification and clustering, information retrieval, query and question answering, ranking in web search engines, plagiarism detection and automatic essay scoring.

The Arabic language is an important language; according to United Nations Educational, Scientific and Cultural Organization (UNESCO), Arabic is one of the six official languages with more than 422 million speakers in the Arab world and used by more than 1.5 billion Muslims. Notwithstanding, automatic processing of Arabic language is not an easy task due to several reasons. Arabic is a very rich derivative and inflectional language; with one lemma it is possible sometimes to build a huge number of different words with varying meanings (Farghaly & Shaalan, 2009). Diacritical marks are used to differentiate between two similar words in writing and different in meaning (same characters but different diacritical marks), which are also called Tashkil or Harakat. Most available text on the web and on digital resources appear without Tashkil, and hence make it harder to resolve disambiguation. Arabic exists in different forms, the old historical Arabic, which is also known as Classic Arabic, Modern Standard Arabic (MSA) which is used nowadays in formal writing, including newspapers and books, and finally dialectal Arabic which is the slang language spoken informally in everyday life and is different from country to another. All of these and other characteristics make Arabic NLP a real challenging task. Compared to the English language, Arabic has modest resources to use in NLP and related fields, and is also considered one of the low-resources languages according to the Association for Computational Linguistics (ACM). Researchers who are interested in low-resource languages such as Arabic lack adequate tools and data sets compared to the English language.

Since 2012, STS was one of the main tasks in SemEval event until 2017. SemEval (2021) is a workshop concerned with the advancement of semantic analysis in NLP and is sponsored by Special Interest Group on the Lexicon (SIGLEX) of ACM.

This study is the first to investigate the effect of stemming on semantic text similarity at the **sentence level** for Arabic language. STS at sentence level is crucial to many applications in contrast to the word and document levels. Many applications rely on computing the similarity between two sentences. Examples are machine translation, generation, summarization, question answering, automatic short answer grading and dialog and conversational systems. Similar research papers studied the effect of stemming on semantic similarity at the word level (Froud, Lachkar & Ouatik, 2012a), and at the document level (Froud et al., 2010), but in the document clustering context. To investigate the effect of stemming and lemmatization on Arabic sentences, different Arabic stemming and lemmatization algorithms are adopted with a total of 10. Before that, standard training and testing data sets are used from SemEval-2017 international workshop for Task 1, Track 1 (Arabic–Arabic).

Also, traditional machine learning algorithms are usually used in text mining and processing. Such algorithms are: SVM with linear kernel, SGD and NB. These algorithms are applied to the STS problem. Different features are extracted and used in the study using String and Character-Based, Statistical-based and Distance-Based similarity measures (Gomaa & Fahmy, 2013). To extract features from data, then three representations are used: Term-Frequency Inverse Document Frequency (TF-IDF) vectors, Word Embedding vectors and the text of the data sets. Word Embedding vectors are used to represent words in sentences. Word embedding vectors usually contain semantic and syntactic features of a word according to its context in a sentence or document (Mikolov et al., 2013).

Compared to the original text, most of the used Arabic stemming algorithms in this study appear superior. Generally, the best on average was ARLSTem and Farasa light stemmers, Farasa and Qalsadi lemmatizers and Tashaphyne heavy stemmer. Hence, it is evident that stemming improves the performance of text similarly for Arabic language at the sentence level. The rest of the paper is organized as follows: next section discusses the related work, after that the adopted data sets are introduced, then the methodology used in the study is explained, then next section illustrates and discusses the results, and finally the paper is concluded.

## Related work

Semantic text similarity aims to determine the degree of likeness or closeness of two pieces of texts. For achieving this task, several similarity measurement methods have been developed. These have become the bases for many natural language processing applications. Examples of such applications are information retrieval, text classification, word sense disambiguation and plagiarism detection. Generally, the research community adopted two typical approaches of text similarity: lexical and semantic (Gomaa & Fahmy, 2013; Wang & Dong, 2020). Lexical similarity means string-based similarity where two texts are similar lexically if they have a similar character sequence whereas semantic similarity indicates similar meaning among texts even they have different words (Farouk, 2019). The methods developed for measuring semantic text similarity at word, sentence and documents levels of Arabic and English texts are reviewed and compared by Alian & Awajan (2020). This paper focuses on semantic similarity of Arabic texts at sentence level and explores mainly the effect of using stemming and lemmatization on semantic text similarity.

Preprocessing is a key task in semantic text similarity process. Stemming is an important technique adopted for preprocessing texts due to the fact that it reduces feature space and improves performance of the similarity process (Alhaj et al., 2019; Almuzaini & Azmi, 2020).

Stemming effect has been studied and applied to different domains of NLP and computation linguistics. This includes document categorization (Alhaj et al., 2019; Almuzaini & Azmi, 2020), information retrieval (Zeroual & Lakhouaja, 2017; Alnaied, Elbendak & Bulbul, 2020), automatic essay scoring (Al-Shalabi, 2016), and sentiment analysis (Al-Saqqa, Awajan & Ghoul, 2019). In all these studies it has been reported that stemming and lemmatization improves the performance of the resulted models.

Al-Ramahi & Mustafa (2012) introduced the importance of stemming in text similarity process and investigated the application of n-gram based matching techniques for measuring similarity of Arabic text documents. The authors reported that word-based bi-gram technique using Cosine similarity provides better accuracy rates than both word-based and whole document-based bi-gram technique using Dice similarity coefficient for Arabic text documents. However, this work neither shown empirically the effect of using stemming on semantic text similarity nor investigated sentence based semantic similarity.

Alhaj et al. (2019) studied the impact of stemming techniques on Arabic document classification. Three stemmers were compared, ISRI (Taghva, Elkhoury & Coombs, 2005), ARLStem (Abainia, Ouamour & Sayoud, 2017) and Tashaphyne (Zerrouki, 2010). Three typical classifiers are used, NB, SVM and K-Nearest Neighbors (KNN). The experiments reported that SVM outperformed NB and KNN with Micro-F1 value of 94.64% when using the ARLStem stemmer. However, this work has focused on the impact of stemming on document categorization rather than semantic text similarity.

Humble work has been performed for the semantic similarity of Arabic sentences (Alian & Awajan, 2020). These works have used three different approaches. First, hybrid similarity approach that uses semantic similarity measure, Cosine similarity measure and N-gram (Kadhem & Abd Alameer, 2017). Second, hybrid feature-based approach that uses lexical, semantic and syntactic-semantic knowledge using Jaccard coefficient, Cosine similarity, and Lexical Markup Framework (LMF) standardized dictionaries (Wali, Gargouri & Hamadou, 2017). Lastly, word embedding using IDF and Part-of-Speech (POS) tagging weighting methods (El Moatez Billah Nagoudi, 2017) and deep learning using Convolutional Neural Networks (CNNs) and Long-Short Term Memory (LSTM) Mahmoud & Zrigui (2019). However, all these works have not studied the effect of stemming on semantic text similarity.

So far, the research studies presented by Froud et al. (2010) and Froud, Lachkar & Ouatik (2012b) are the only works that have investigated the effect of using stemming on semantic similarity of Arabic text. Froud et al. (2010) investigated diverse similarity measures with document clustering and they applied stemming to words which have reduced documents representation and provided fast clustering. Froud, Lachkar & Ouatik (2012b) tested the effect of using stemming and light stemming on the semantic similarity between Arabic words. The similarity is measured by Latent Semantic Analysis (LSA) and computed by using different measures. They have used Euclidean Distance, Cosine Similarity, Jaccard Coefficient and the Pearson Correlation Coefficient. The obtained results show that light stemming outperforms stemming approach. However, these two research papers are implemented at document level and word level respectively and not on sentence level. Also, Froud et al. (2010) is concerned with document clustering rather than semantic text similarity.

SemEval-2017 task 1 is an event held for measuring the STS of sentences. STS tasks in SemEval have been held from 2012 to 2017 (Cer et al., 2017). In SemEval-2017, STS shared task concentrated on the evaluation of semantic similarity between monolingual and cross-lingual sentences in Arabic, English, Spanish and Turkish which were organized into a set of six tracks. There were 49 participants for track 1, Arabic (ar–ar). Similarity for sentence pairs is measured as a real-valued score ranging from 0 for dissimilar sentences to five for equivalent sentences. Performance is measured by the Pearson correlation coefficient between machine scores and human judgments. The best performance achieved for track 1 is Pearson correlation of 75.43% performed by Wu et al. (2017). It is worth mentioning that the effect of stemming on STS has not been investigated on all tracks including track 1 in SemEval-2017 task 1. However, Meng et al. (2017) were the only work in SemEval-2017 task 1 that has demonstrated the effect of lemmatization on all translated sentence pairs of text to English using Google translator.

In fact, no work has studied the effect of using stemming and lemmatization on semantic text similarity for Arabic sentences. This limitation was the motivation for the contribution of this paper.

## Data

Arabic language exists in three forms: classical, MSA, and dialectal. As described in the introduction, MSA is the one used nowadays in media, newspapers and books. To make it clear, the following are examples of the three forms:

- Classical Arabic: 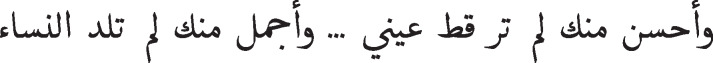
- MSA: 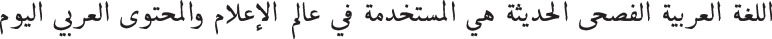
- Dialectal (Levantine): 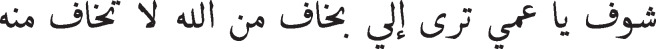

In order to investigate the effect of stemming on STS for Arabic language at sentence level, standard data sets from SemEval2017 (Cer et al., 2017) are adopted for both training and testing as Tables 1 and 2 show. The form of language used in these data sets is MSA. The training sentence pairs sum to 1,081 while the gold testing pairs are 250. The training data is collected from different sources (Cer et al., 2017):

**Table 1:** Arabic training data.

Year	Data set	Pairs	Source
2017	MSRpar	510	Newswire
2017	MSRvid	368	videos
2017	SMTeuroparl	203	WMT eval.

**Table 2:** Arabic gold test data.

Track	Language(s)	Pairs	Source
1	Arabic (ar–ar)	250	SNLI

- MSRpar: Microsoft Research Paraphrase Corpus.
- MSRvid: Microsoft Research Video Description Corpus.
- SMTeuroparl: WMT2008 development dataset (Europarl section).

While the gold testing data are taken from the Stanford Natural Language Inference (SNLI) Corpus.

Training and testing data sets include pairs of sentences with corresponding human similarity score. The scoring system used is on a scale from 0 to 5 according to Table 3 (Agirre et al., 2013). The table explains the meaning of every score along with an example in English and Arabic languages. Please note that English and Arabic example are different.

**Table 3:** Similarity scores with explanations, English examples from Agirre et al. (2013), and Arabic examples from Gold Test Data of SemEval-2017.

Score	Meaning, English and Arabic Examples
5	***The two sentences are completely equivalent, as they mean the same thing*.**
	The bird is bathing in the sink.
	Birdie is washing itself in the water basin.
	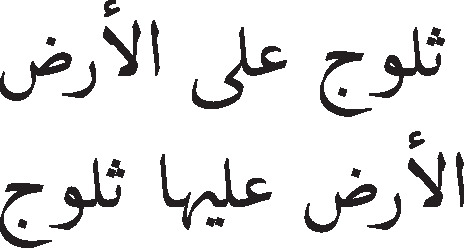
4	***The two sentences are mostly equivalent, but some unimportant details differ*.**
	Two boys on a couch are playing video games.
	Two boys are playing a video game.
	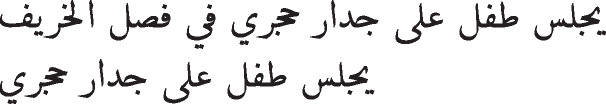
3	***The two sentences are roughly equivalent, but some important information differs/missing*.**
	John said he is considered a witness but not a suspect.
	“He is not a suspect anymore.” John said.
	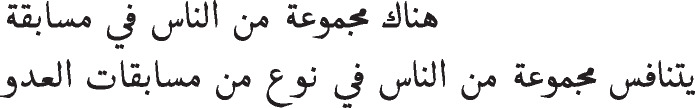
2	***The two sentences are not equivalent, but share some details*.**
	They flew out of the nest in groups.
	They flew into the nest together.
	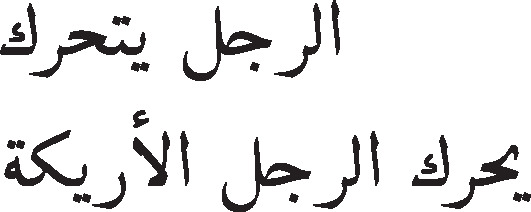
1	***The two sentences are not equivalent, but are on the same topic*.**
	The woman is playing the violin.
	The young lady enjoys listening to the guitar.
	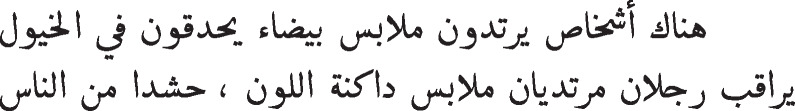
0	***The two sentences are completely dissimilar*.**
	The black dog is running through the snow.
	A race car driver is driving his car through the mud.
	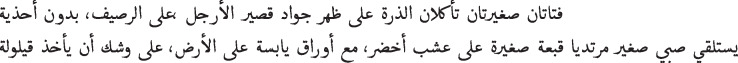

## Methodology

The methodology adopted in this study is divided into four parts. They will be listed here briefly before being explained in the following subsections:

1. Select stemming algorithms and prepare new training and testing text documents after preprocessing text and applying the selected stemming algorithms.
2. Choose the machine learning algorithms to use in the study and choose the baseline for experiments.
3. Select the features to use in experiments by applying String and Character-Based, Term-based and Distance-Based similarity measures into three representations of data sets: TF-IDF vectors, Word Embedding vectors and text of the data sets.
4. Setup experiments by applying the chosen machine learning algorithms to the original and prepared text documents using the selected features.

**Figure 1:**
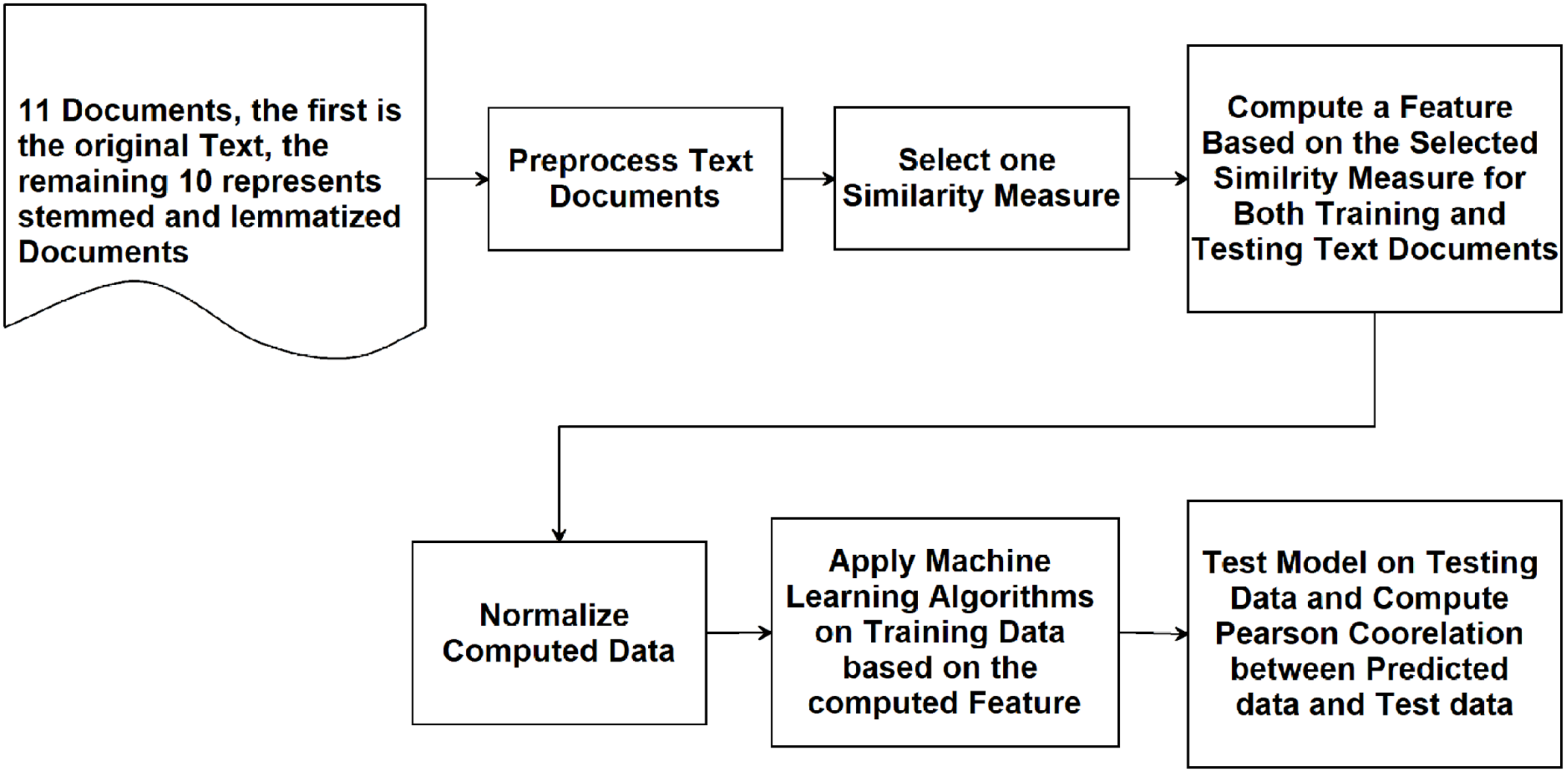
Flowchart illustrating the methodology adopted in this study.

The main steps of the methodology are illustrated in the flowchart given in Fig. 1. This flowchart represents the process that will be repeated for all similarity measures according to each representation of the text documents: TF-IDF vectors, Word Embedding vectors, and text as explained in Table 4.

**Table 4:** Documents and stemming Algorithms used in the study.

Document	Description
Original Text	Original Text of SemEval-2017
AlKhalil Stems	Stems of Original Text using AlKhalil’s stemmer (Boudchiche et al., 2017)
ARLSTem Stems	Stems of Original Text using Arabic light stemmer (Abainia, Ouamour & Sayoud, 2017)
Assem Stems	Stems of Original Text using Assem’s stemmer (Chelli, 2018)
Farasa Lemmas	Lemmas of Original Text using Farasa Algorithm (Abdelali et al., 2016)
Farasa Stems	Stems of Original Text using Farasa stemmer (Abdelali et al., 2016)
ISRI Stems	Stems of Original Text using ISRI stemming (Taghva, Elkhoury & Coombs, 2005)
Khoja Roots	Roots of Original Text using Khoja Algorithm (Khoja & Garside, 1999)
Qalsadi Lemmas	Lemmas of Original Text using Qalsadi Algorithm (Zerrouki, 2012)
Tashaphyne Roots	Stems of Original Text using Tashaphyne Algorithm (Zerrouki, 2010)
Tashaphyne Stems	Stems of Original Text using Tashaphyne stemmer (Zerrouki, 2010)

### Prepare documents

There exist several stemming algorithms for Arabic language. Also, there are mainly two types of stemmer algorithms: light stemmers and heavy stemmers (root extractors). Besides, lemmatization algorithms may improve the performance results understudy, lemma is defined as the original of a word. All these three methods are expected to reduce the dimension space of features and reduce similar words in meaning but different in morphology to the same stem, root, or lemma, and hence increase the similarity. Similar studies at word and document levels (Froud et al., 2010), used two stemming algorithms, one light and the other is heavy. Therefore, to broaden the applicability of this study, from existing algorithms in the literature, 6 light stemmers, 2 heavy stemmers and 2 lemmatization algorithms are selected which sum to 10. This study uses 11 text documents including the original text, they are listed in Table 4.

The stemming and lemmatization algorithms are applied to both training and testing data sets using python where packages are available for some algorithms. For other stemming algorithms, only java implementation is available, and then the jar files are called from within python and executed. A couple of algorithms have only online web service; hence a python request is executed to collect the stem of a word.

Elementary preprocessing algorithm is used in preparing all data sets by simply keeping only Arabic letters. It is necessary to mention that stop words have not been removed, because by removing them the performance become worse in all experiments. The preprocessing algorithm is as following:

1. Remove punctuation marks.
2. Remove non-Arabic characters including English.
3. Remove numbers.

### Choose ML algorithms

Different machine learning algorithms are considered for creating models for the STS scheme. At the beginning, all the following algorithms are used in all experiments: SVM, SGD, Linear Regression, Bayesian Regression, Decision Trees, Random Forests, Bagging, KNN, Gradient Boosting, Ada Boosting and XG-Boosting. All are abandoned except those with the best performance: SVM, SGD and NB. These are combined in a fourth model using Ensemble learning. These algorithms typically give good results when used in text processing problems, because they are usually linearly separable. Also, most of the algorithms used in this study are already utilized in some research papers participated in SemEval-2017 Task 1 competition, and this make results of this study comparable with their results.

The baseline used here is the Pearson correlation for cosine similarity. This is computed between each pair of the gold test sentences using Binary encoding, TF-IDF encoding and word embedding vectors. The Pearson Correlation score for these three representations was: 62.32%, 63.04% and 55.66% respectively. Please note that the Pearson correlation for cosine similarity between Binary Encodings of gold test sentences is also used as a base line in SemEval-2017.

### Select features

To judge the effect of stemming reliably, different features are computed according to different similarity measures, and applied to three main feature types: TF-IDF vectors, word embedding vectors-trained using deep learning models and text sentence pairs. The similarity measures used belongs to string and character based, statistical based and distance-based measures. Most of these features are used in SemEval-2017 top-wining studies. Both statistical and distance based computed features are used in TF-IDF and word embedding vectors, while string and character-based features are computed directly using the text of the data sets. The features and measures are listed in Table 5. In the case of word embedding vectors, the FastText model for Arabic language is used (Bojanowski et al., 2016) with dimensionality of 300. Each sentence of the pairs will be replaced by a vector representing the average of the word embedding vectors for the words of that sentence.

**Table 5:** Features and similarity measures used in the study.

Type	Measure	Applied to
Statistical	Correlation Coefficient	TF-IDF and Word Embedding vectors
	Pearson Coefficient	TF-IDF and Word Embedding vectors
	Cosine Measure	TF-IDF and Word Embedding vectors
	Kendall’s tau Coefficient	TF-IDF and Word Embedding vectors
Distance	Euclidean distance	TF-IDF and Word Embedding vectors
	Manhattan distance	TF-IDF and Word Embedding vectors
String-Character	Character Bi-grams Overlap	Text of the data sets
	Character Tri-grams Overlap	Text of the data sets
	Common words Overlap	Text of the data sets
	Jaccard Coefficient	Text of the data sets

It is important to observe that only bi-grams and tri-grams overlap measures are used. This is because 4-grams, 5-grams, and higher grams give worse results in experiments. This makes sense as the average number of characters for Arabic words is about 5–6 (Alotaiby, Alkharashi & Foda, 2009). Therefore, 4-grams and higher might not retrieve on average more than the word itself and sometimes two to three sequences. Hence, this makes the size of the overlapped sequences minimal.

We define Character n-Grams overlap similarity measure as a set-based measure, this an experiment deduced measure rather than an intuition one:

(1) $$nGrams-Overlap=\frac{|S_{1}\cap S_{2}|}{mean(|S_{1}|,|S_{2}|)}$$

where *S*_1_ and *S*_2_ are n-Gram sequences for both sentences 1 and 2, respectively. This measure improves the performance of Pearson correlation results in matching experiments. The average of the length of tokens for both sentences gives the best results compared to min and max functions.

Jaccard Coefficient for text similarity can be defined as:

(2) $$Jaccard-Overlap=\frac{|L_{1}\cap L_{2}|}{|L_{1}\cup L_{2}|}$$

where *L*_1_ and *L*_2_ are the set of unique terms for both sentences 1 and 2, respectively.

We define the common words overlap measure as:

(3) $$CommonWords-Overlap=\frac{|L_{1}\cap L_{2}|}{mean(|L_{1}|,|L_{2}|)}$$

Again, this similarity measure is an experimental rather than intuitional. This improves the performance of Pearson correlation results in matching experiments. Again, the average of the length of tokens for both sentences gives the best results compared to min and max functions. Compared to Jaccard-overlap which is defined in Eq. (2), the common-words overlap measure improves results by around 2–3% in Pearson Correlation.

### Setup experiments

Three experiments will be set up to investigate the effect of stemming on Arabic STS task. According to the features selected in previous section and as described in Table 5, the following experiments will be used in the study:

1. Experiment I: Apply ML algorithms to features computed by statistical and distance-based similarity measures and using TF-IDF vectors representation of the data sets.
2. Experiment II: Apply ML algorithms to features computed by statistical and distance-based similarity measures and using Word Embedding vectors representation of the data sets.
3. Experiment III: Apply ML algorithms to features computed by string and character-based similarity measures and directly using text of the data sets.

Each experiment is repeated to compute one feature at a time based on one similarity measure according to Table 5. For example, Experiment I will be executed 8 times: 4 times for statistical measures and the fifth for all together, 2 times for distance based measures and the third for both measures together. All these experiments will run on all ML algorithms stated in this section.

Moreover, one extra experiment is setup to compute the Pearson Correlation for different combinations of similarity measures that will give best results. In addition to several experiments to choose the best ML Algorithm, best similarity measures, …etc. It is vital here to stress that the main theme of the paper is to study the effect of stemming and lemmatization on STS problem and not to improve results by providing new methodology. However, such experiments support the main theme of the paper by showing how using simple methodology, which is based on stemming and lemmatization, would improve dramatically the performance in STS problem.

Please notice that in all figures, tables and text in this study, performance is reported by as Pearson’s r multiplied by 100 values.

## Results and discussion

**Figure 2:**
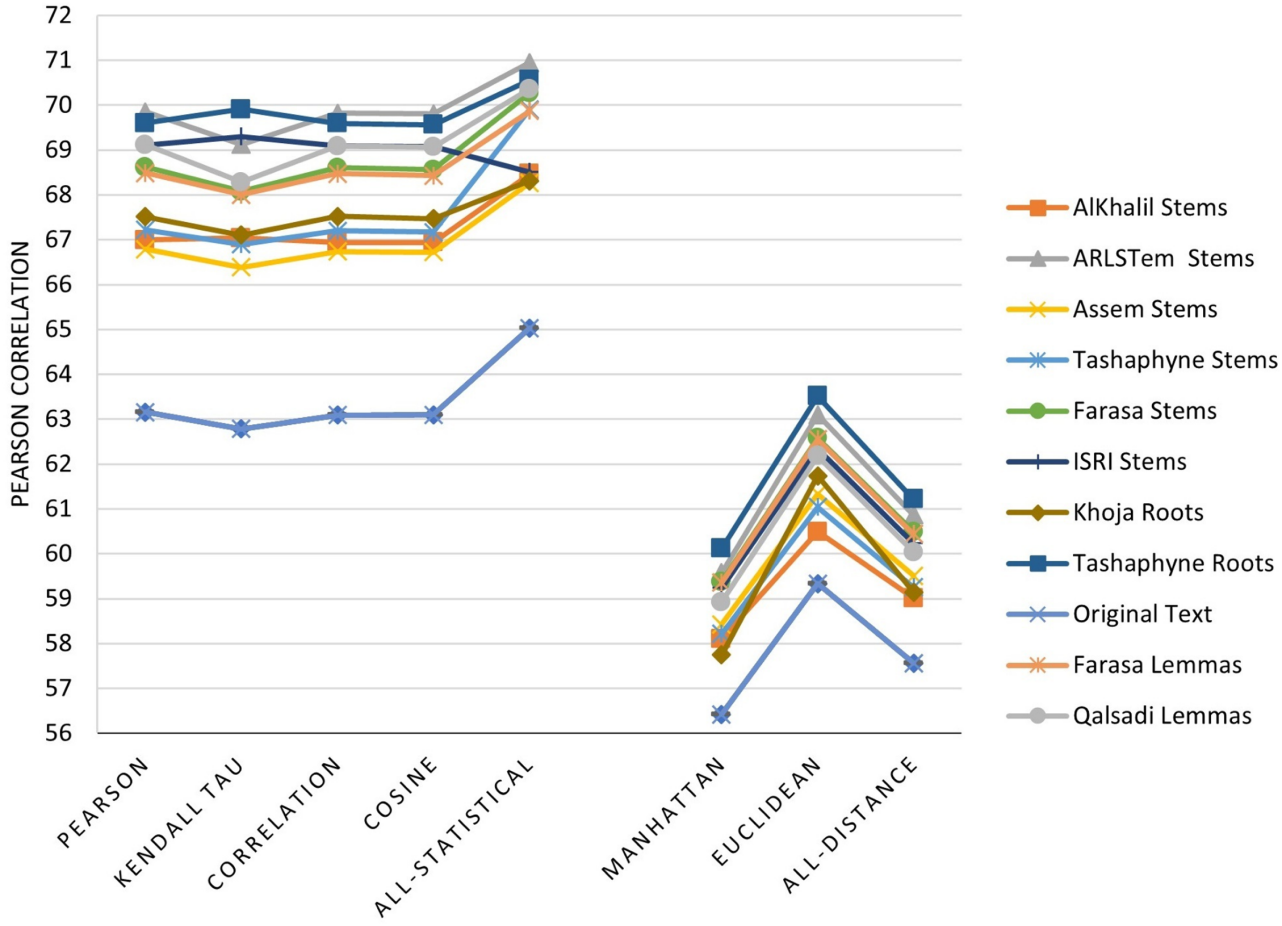
Pearson correlation for similarity measures applied to TF-IDF vectors.

**Figure 3:**
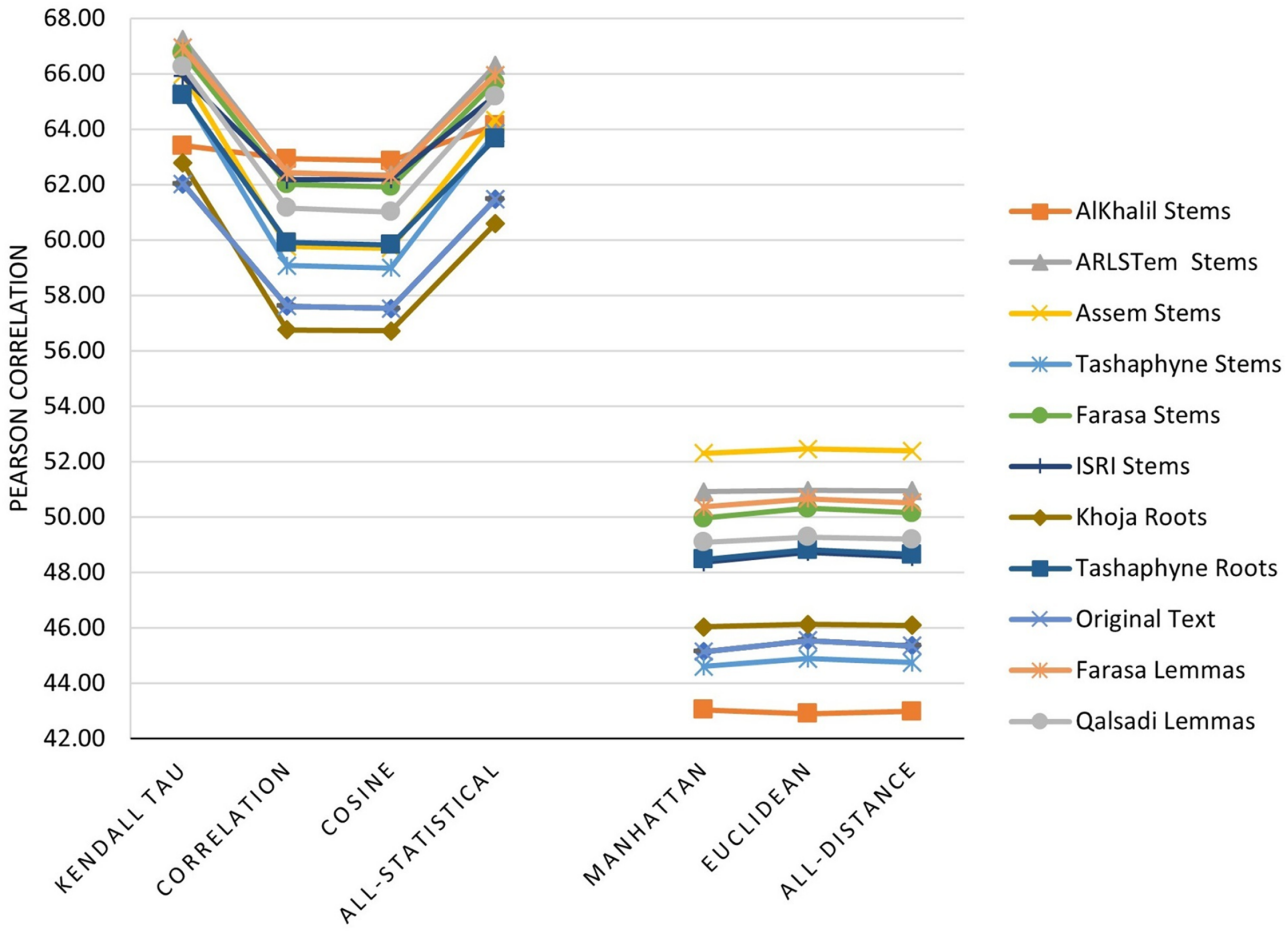
Pearson correlation for similarity measures applied to Word Embedding vectors.

**Figure 4:**
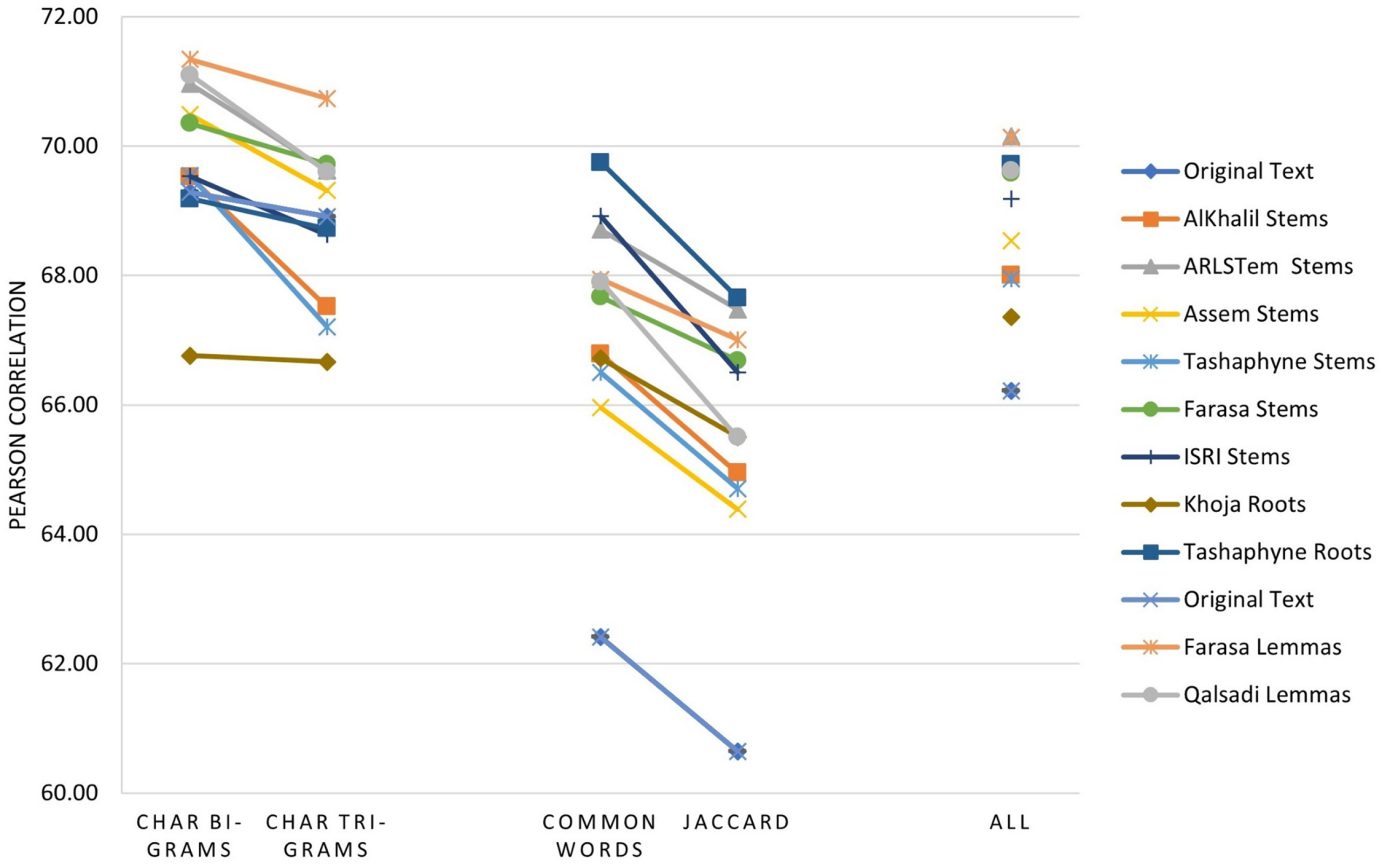
Pearson correlation for similarity measures applied immediately to the text of the data sets.

The main three experiments described in the previous section are executed and results are depicted in Figs. 2–4. Figure 2 depicts results of Pearson correlation using similarity measures applied to TF-IDF vectors. Figure 3 depicts results using similarity measures applied to Word Embedding vectors. Lastly, Fig. 4 depicts results using string and character based similarity measures applied immediately to the text of the data sets. These are applied to 11 documents as indicated previously.

**Figure 5:**
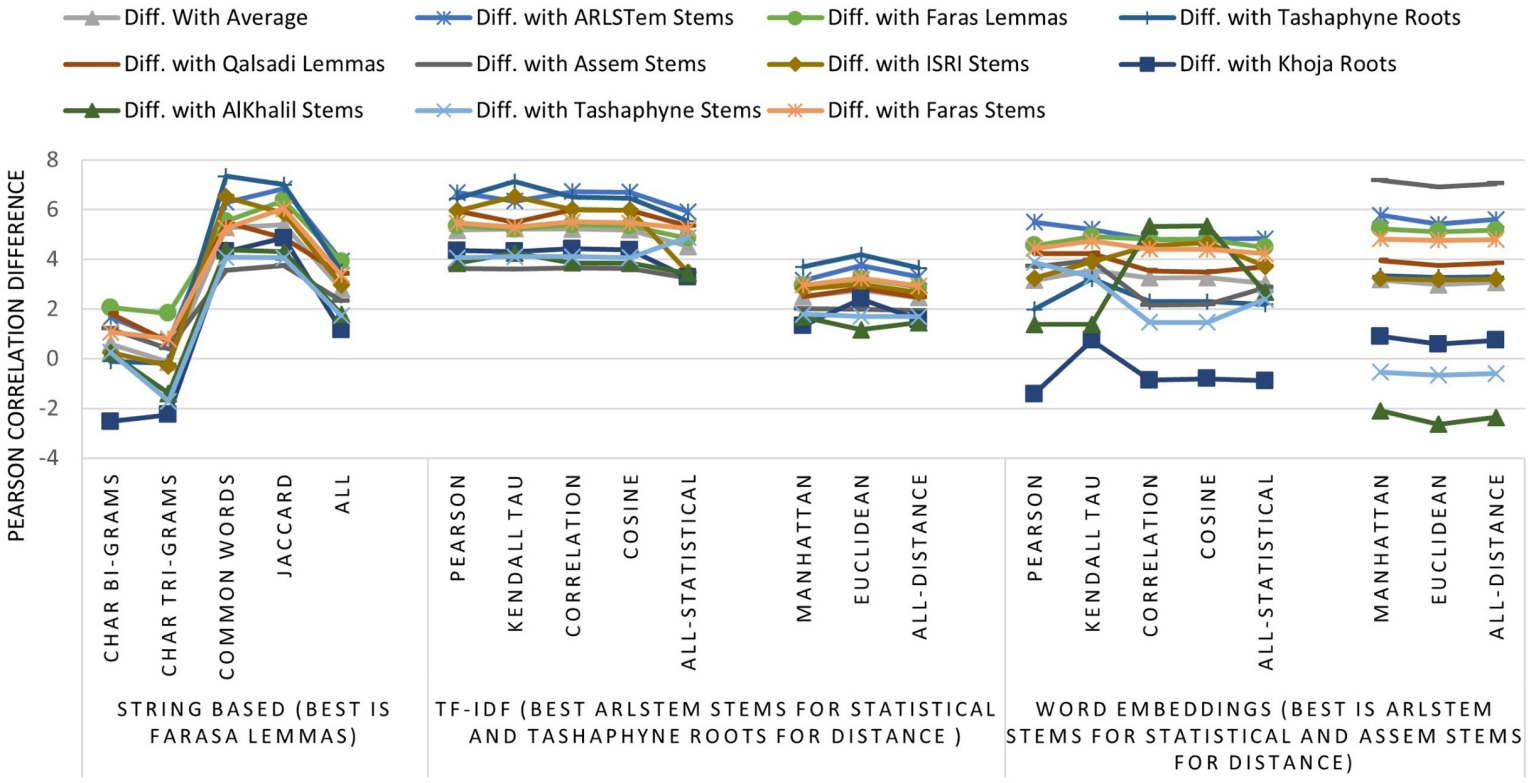
Pearson correlation Improvements after applying Stemming Algorithms for all similarity measures applied to Text of the dataset, TF-IDF vectors, and Word Embedding vectors.

The results on all experiments show spectacular improvement on Pearson correlation after applying stemming and lemmatization algorithms as depicted in Fig. 5. For statistical measures and using TF-IDF representations, the enhancement in Pearson correlation is in the range of 3.24–7.13% with best results, in order, achieved using: ARLSTem stems, Tashaphyne roots, ISRI stems and Qalsadi lemmas. However, using Word Embedding representations the statistical similarity measures achieve lower results with the range of −1.42–5.5%, where the negative value means that some stemming algorithms, which is in this case Khoja heavy stemmer gives results worse than when using the original text. The best results here are achieved when using: ARLSTem stems, Farasa lemmas, Farasa stems and Qalsadi lemmas. Note that AlKhalil stems sometimes gives superior results and other times lowest results, therefore it is not considered among the best.

On the other hand, for distance similarity measures using TF-IDF representations, the enhancement in Pearson correlation was in the range 1.16–4.18%. Ordered best algorithms in this case was: Tashaphyne heavy stemmer, ARLSTem stemmer, Farasa stemmer and Farasa lemmatizer. Whilst using Word embedding representations, distance similarity measures give results in the range of −2.64–7.17%. Where negative value in this case was for the worst results using AlKhalil Stemmer. The best results here attained using Assem stemmer, ARLSTem stemmer, Farasa lemmatizer and Farasa stemmer.

Lastly, for string and character similarity measures and using text representation, the improvement in Pearson correlation was in the range of −2.53–7.34%. Where negative difference yielded using Khoja heavy stemmer. In this case, results are reluctant using string and character-based similarity measures. Therefore, best algorithms are ordered based on using all features resulted from choosing the four measures together. Hence, the best algorithms in order are: ARLSTem stemmer, Farasa Lemmatizer, Tashaphyne heavy stemmer and Qalsadi lemmatizer.

Note from Fig. 5 that for statistical based measures; Kendall’s tau gives the best improvement using TF-IDF representations, while it was Pearson coefficient in the case of using Word embedding representations. For distance-based measures, the best measure was Euclidean for TF-IDF vectors, where it was Manhattan for Word embedding vectors. For String based measures, the best improvement occurs when using common words measure defined in Eq. (3).

Many experiments are executed to find the best combination of features that will give the best performance for the model. It is found that the best results are achieved with character bi-gram along with cosine measure using TF-IDF vectors. The results are shown in Table 6. Please notice that in one experiment, all features in the study are used. The results were worst compared with the two best features. Note that the best results are achieved when using Qalsadi Lemmas, ARLSTem Stems and Farasa Lemmas with around 71.5% of Pearson correlation. Compared to the best results achieved by SemEval-2017 winning team (Tian et al., 2017); this is comparable as the results of this study use only two features compared to 67 features used in Tian et al. (2017) which achieved 75.43%.

**Table 6:** Results on Gold Test data for the best two features: character bi-gram and cosine Bold entries indicate the Pearson Correlation values for the best three stemming and lemmatization algorithms.

Document	SVM (%)	SGD (%)	NB (%)	Ensemble (%)
Original Text	66.62	66.75	69.04	69.18
AlKhalil Stems	69.34	69.39	69.76	69.85
**ARLSTem Stems**	**71.55**	71.55	70.43	70.69
Assem Stems	69.45	69.55	70.64	70.69
**Farasa Lemmas**	70.71	70.76	71.51	**71.54**
Farasa Stems	70.58	70.62	70.61	70.71
ISRI Stems	70.49	70.50	70.03	70.13
Khoja Roots	68.47	68.48	64.98	65.45
**Qalsadi Lemmas**	71.33	71.37	71.51	**71.60**
Tashaphyne Roots	70.61	70.61	70.13	70.20
Tashaphyne Stems	69.47	69.49	69.76	69.84

Note that the effect of lemmatization and stemming is clear if Pearson correlation for the original text is compared to the best algorithms with enhancement of about 5%. Most results show that stemming algorithms improve the Pearson correlation compared to original text. However, in some cases such as Khoja heavy stemmer results become worse. This might be because heavy stemmer reduces tokens to their roots, and in many cases different words which have different meanings might have the same root. Notwithstanding, they may have at the same time different stems or lemmas. Another reason behind this might be the accuracy of stemmers. For example, while Khoja gives bad results, as a heavy stemmer, Tashaphyne still gives much better results in comparison. Another reason might be the accuracy of the algorithm. While two words should have the same stem, some stemmers would fail to retrieve the same stem. Instead, it gives two different stems and hence increase the dimensionality of the data representing each sentence, and as a result will reduce similarity.

Among all results, those given by ARLSTem and Farasa light stemmers on average are the best when being applied to TF-IDF representations for the data sets. In addition, Farasa and Qalsadi Lemmatizers also gave particularly good results as lemmas might decrease the dimensionality of data representing each sentence, and hence would increase similarity.

## Conclusion

Semantic text similarity (STS) is considered one of the vital tasks in NLP and computational linguistics. In this study, the effect of stemming and lemmatization is studied on STS for Arabic sentences. Many stemming and lemmatization algorithms for Arabic are considered in this study. These are applied to a standard data set for STS problems, where data sets used in SemEval-2017 are utilized here. At the same time, different similarity measures are adopted including string and character, statistical and distance-based similarity measures. These are applied to three types of data representations: TF-IDF vectors, Word embedding vectors and the text itself. Different experimental setups are implemented to achieve the aims of the study.

Results show superior improvement on Pearson correlation when using stemming and lemmatization algorithms in measuring similarity between Arabic sentences. This study recommends using the following algorithms for Arabic STS on sentence level: ARLSTem and Farasa light stemmers, Farasa and Qalsadi Lemmatizers, and Tashaphyne heavy stemmer. Notwithstanding, Khoja heavy stemmer and AlKhalil light stemmers, in general, gave the worst results. Also, the results show that the best data representation for data sets is the TF-IDF vectors. Furthermore, the study redefines Jaccard overlap as a “common words overlaps” and experimentally improves result of Pearson correlation by 2–3%. Moreover, a search for the best combination of features based on similarity measures is performed; the best combination was to use bi-grams overlap and cosine similarity for TF-IDF vectors of sentences.

Future work might investigate the effect of stemming and lemmatization on STS task using deep learning models, although the results of this study suggest that TF-IDF representations give much better results compared to word embedding representations. Word embedding vectors are trained and evolved from deep learning models. However, training STS data after applying stemming and lemmatization algorithms using deep learning models might improve similarity results.

## Supplemental Information

10.7717/peerj-cs.530/supp-1 Supplemental Information 1 All 11 documents used in experiments along with human score files for both training and testing data.

10.7717/peerj-cs.530/supp-2 Supplemental Information 2 A python program that computes Pearson Correlation for String and character based similarity measures.

10.7717/peerj-cs.530/supp-3 Supplemental Information 3 A python program that computes Pearson Correlation for Statistical and distance based similarity measures using Word Embedding vectors.

10.7717/peerj-cs.530/supp-4 Supplemental Information 4 A python program that computes Pearson Correlation for Statistical and distance based similarity measures using TF-IDF vectors.

## Additional Information and Declarations

### Data Availability

The following information was supplied regarding data availability: The raw data and stemmed files and code for experiments are available in the Supplemental Files. The raw testing and training data are also available at GitHub https://github.com/malhawarat/STS_Ara_Stem_Effect.
